# Retraction: Selected thiadiazine-thione derivatives attenuate neuroinflammation in chronic constriction injury induced neuropathy

**DOI:** 10.3389/fnmol.2026.1827289

**Published:** 2026-03-17

**Authors:** Frontiers Editorial Office

The Journal retracts the 2021 article cited above.

Following publication, concerns were raised regarding the integrity of the images in the published figures. Image duplication concerns were identified in Figure 6E, and between Figures 4D and 7F.

The authors failed to provide a satisfactory explanation during the investigation, which was conducted in accordance with Frontiers' policies. As a result, the data and conclusions of the article have been deemed unreliable and the article has been retracted.

This retraction was approved by the Chief Editors of Molecular Neuroscience and the Chief Executive Editor of Frontiers. The authors agree to this retraction.

